# Heterotopic Transcallosal Projections Are Present throughout the Mouse Cortex

**DOI:** 10.3389/fncel.2017.00036

**Published:** 2017-02-21

**Authors:** Alexandra Chovsepian, Laura Empl, Daphne Correa, Florence M. Bareyre

**Affiliations:** ^1^Institute of Clinical Neuroimmunology, Biomedical Center and University Hospital, Ludwig-Maximilians Universität MünchenMunich, Germany; ^2^Munich Cluster of System Neurology (SyNergy), Ludwig-Maximilians Universität MünchenMunich, Germany

**Keywords:** anterograde tracing, corpus callosum, retrograde tracing, transcallosal axons, clearing

## Abstract

Transcallosal projection neurons are a population of pyramidal excitatory neurons located in layers II/III and to a lesser extent layer V of the cortex. Their axons form the corpus callosum thereby providing an inter-hemispheric connection in the brain. While transcallosal projection neurons have been described in some detail before, it is so far unclear whether they are uniformly organized throughout the cortex or whether different functional regions of the cortex contain distinct adaptations of their transcallosal connectivity. To address this question, we have therefore conducted a systematic analysis of transcallosal projection neurons and their axons across six distinct stereotactic coordinates in the mouse cortex that cover different areas of the motor and somatosensory cortices. Using anterograde and retrograde tracing techniques, we found that in agreement with previous studies, most of the transcallosal projections show a precise homotopic organization. The somata of these neurons are predominantly located in layer II/III and layer V but notably smaller numbers of these cells are also found in layer IV and layer VI. In addition, regional differences in the distribution of their somata and the precision of their projections exist indicating that while transcallosal neurons show a uniform organization throughout the mouse cortex, there is a sizeable fraction of these connections that are heterotopic. Our study thus provides a comprehensive characterization of transcallosal connectivity in different cortical areas that can serve as the basis for further investigations of the establishment of inter-hemispheric projections in development and their alterations in disease.

## Introduction

The mammalian neocortex is a highly complex layered structure containing a large number of cell types that need to act in a coordinated fashion to enable the execution of elaborate cognitive and behavioral tasks. The ability to perform those complex tasks relies on the highly organized connections between those different neuronal subtypes (Schoenemann et al., 2005). Transcallosal neurons, inter-hemispheric commissural large pyramidal cells, whose myelinated axons form the corpus callosum and integrate information between the two cortical hemispheres (Aboitiz and Montiel, 2003; Fame et al., 2011; Zhou et al., 2013), are a good example of this organization pattern. The anatomical studies of this neuronal population have relied on anterograde and retrograde labeling techniques to identify the cells of origin of the corpus callosum and to reveal their cortical organization in rats, cats or monkeys (Jacobson and Trojanowski, 1974; Wise and Jones, 1976). Later studies used biotinylated dextran amines (BDAs) to describe their organization in more detail and provide a more refined understanding of global connectivity in the cortex (Yorke and Caviness, 1975; Aboitiz and Montiel, 2003; Schüz et al., 2006). From these studies we know, that the cells of origin of the corpus callosum are principally located in cortical layers II/III and to a lesser extent in layer V (Fame et al., 2011). Further, their connections extend homotopically to the contralateral hemisphere (Yorke and Caviness, 1975; Aboitiz and Montiel, 2003; Garcez et al., 2007; Fame et al., 2011). So far however no systematic analysis of these connections—using identical anatomical tracers and techniques—has been performed that allows us to compare the organization of transcallosal connections in different functional regions of the mouse cortex including the primary motor and somatosensory cortices. Such a systematic analysis of inter-hemispheric connectivity is important as defects in those projections—either innate or due to injury—can lead to disturbed connectivity and have been proposed to contribute to altered cortical processes in autism and related disorders (Egaas et al., 1995; Vidal et al., 2006; Paul et al., 2007; Freitag et al., 2009; McAlonan et al., 2009). However it is for example still unclear, whether specific regional populations of transcallosal neurons are differentially affected by or susceptible to such pathological alterations. A first step to approach this question is to characterize the regional variability of transcallosal connections across the mouse cortex.

To achieve this aim, we have used the retrograde tracer FluoroGold (FG), as well as the anterograde tracer BDA, to respectively label the projection neurons and axonal connections of transcallosal neurons in six distinct cortical locations spanning primary motor and primary somatosensory cortices, including the barrel cortex. In doing so, we were able to investigate whether the homotopic columnar organization of transcallosal neurons and their distribution in cortical layers II/III and V is a blueprint of all transcallosal connections or whether this organization differs depending on the location examined. In particular, we focused on the following questions: (i) Are all transcallosal connections in mice organized homotopically throughout the cortex and how are the terminal fields of transcallosal axons distributed within the contralateral homotopic cortical column? (ii) In which layers are the somata of transcallosal neurons predominantly located? and (iii) What is the regional variability of these connections in terms of the location of the projection cells, their columnar organization, and the contribution of non-homotopic transcallosal projections?

Here we found in agreement with previous studies that most of the transcallosal projections show a precise homotopic organization and that terminal axons of the transcallosal projections span the entire extent of the cortical column. The cells of origin of these connections are primarily located in layer II/III as well as in layer V. However we further found a relevant fraction of these cells in cortical layer IV and layer VI. Comparison of different cortical regions revealed that subtle regional differences exist both with regard to the distribution of transcallosal cell bodies along the cortical column as well as with regard to the precision of their projections. While overall this neuronal population shows a remarkably homogenous organization throughout the cortex, we also found heterotopic connections that are present throughout the mouse cortex and show a comparably higher level of regional variability. Our data support the well-known overall uniformity of interhemispheric connections that might be important for the robust execution of a range of cortical functions but also reveal the presence of regional heterogeneity that might help to mediate their local adaptation.

## Materials and Methods

### Animals

Six to twelve week-old female C57/Bl6 mice (Janvier SAS; *n* = 32) were used for this study. All experimental procedures were performed according to the German guidelines on animal welfare and were approved by local regulatory committees (Regierung von Oberbayern).

### Tracer Injections

Anesthesia was induced by i.p. injections of Midazolam (5.0 mg/kg bodyweight)/Medetomidine (0.5 mg/kg bodyweight)/Fentanyl (0, 05 mg/kg bodyweight) in accordance with local animal welfare recommendations. We confirmed that animals were sufficiently anesthetized by the absence of pedal reflex. In order to characterize the axonal terminal fields of transcallosal neurons, we injected 1.5 μl of the anterograde tracer BDA (10,000 MW; Life Technologies; Reiner et al., 2000; Bareyre et al., 2002, 2004) using a finely pulled glass micropipette (coordinates from Bregma: −1.5 mm; 1.7 mm lateral; 0.3 mm depth to target layers II/ III and 0.6 mm depth to target layer V). The micropipette remained in place for 3 min following the injection to avoid backflow.

To describe the location and distribution of transcallosal projection neurons, we retrogradely labeled them by stereotactically injecting 0.5 μl of (FG; 1% in 0.1 M Cacodylate buffer, Fluorochrome LLC) in the primary motor cortex, the primary somatosensory cortex outside and inside the barrel cortex area. Briefly, a small hole was drilled in the skull and a glass capillary micropipette tip was slowly lowered into the brain tissue before injection. The pipette tip remained 3 min in the brain after the injection was completed to limit backflow and was then removed from the tissue. We used the following injection coordinates with respect to Bregma: rostrocaudal +0.3 mm and −1.5 mm, lateral 1.3 mm, depth 0.3 mm; rostrocaudal +0.3 mm and −1.5 mm lateral 1.7 mm, depth 0.3 mm and rostrocaudal +0.3 mm and −1.5 mm, lateral 3.5 mm, depth 0.3 mm).

After the surgery, mice were allowed to wake up on a heating pad (38°C) and received analgesic treatment with Metacam (0.05 mg/kg, Boehringer Ingelheim) up to 48 h after the procedure. All animals were sacrificed 10 days post-surgery, in order to ensure that the tracer has enough time to travel efficiently to its target location.

### Generation, Production and Stereotactic Injection of Recombinant AAV Vectors

For the tissue clearing experiment, we anterogradely labeled transcallosal axons with an adeno-associated virus (AAV) expressing the yellow fluorescent protein. We generated pAAV-CAG-EYFP (rAAV-EYFP) by inserting EYFP (from pEYFP-N1) into pAAV-CAG-MCS. Recombinant AAV chimeric virions containing a 1:1 ratio of AAV1 and AAV2 capsid proteins were generated as previously described (Grimm et al., 2003; Klugmann et al., 2005; Lang et al., 2013; Jacobi et al., 2015). To anterogradely label transcallosal axons, we pressure injected 0.7 μl of rAAV-CAG- EYFP at two adjacent injection sites into layer II/III of the somatosensory cortex using a finely pulled glass micropipette (concentration 0.6 × 10^12^ genome copies/ml; coordinates from Bregma: −1.5 mm; 1.7 and 1.9 mm lateral, 0.3 mm depth) 10 days prior to brain clearing. The micropipette remained in place for 3 min following the injection to avoid backflow.

### Tissue Preparation for Analysis

Animals were sacrificed with isoflurane and perfused with PBS-Heparin (1:500) solution followed by 4% paraformaldehyde (PFA) in 0.1 M phosphate buffer (PBS). Tissue was post-fixed with 4% PFA for 24 h and subsequently the brains were removed for cutting on a vibratome (Leica). Sections were cut at a thickness of 100 μm for identification of transcallosal neurons and stained free-floating with NeuroTrace 435 (ThermoFischer Scientific; 1:500 in 0.1% Triton PBS) overnight at 4°C. Following incubation, sections were again washed three times in 1 ×PBS for 10 min and finally mounted on slides with VectaShield (Vector Laboratories).

### Imaging and Image Processing

Confocal image stacks of transcallosal axons (anterogradely labeled) and transcallosal projection neurons (retrogradely labeled) were acquired with an Olympus FV1000 confocal microscope equipped with standard filter sets. The images of the retrogradely labeled axons were taken with a 10× objective, zoom 1× and 1024 × 1024 resolution using Kalman filtering and scanning 10 μm of the total section thickness using a *z*-step size of 1 μm. To image anterogradely labeled transcallosal axons and transcallosal projections, confocal image stacks were acquired using a Leica SP8 confocal microscope with a 20× objective, zoom 1× and 1024 × 1024 resolution and a *z*-step size of 1.04 μm. Image stacks were processed using ImageJ software to generate maximum intensity projections. To obtain final images, these maximum intensity projections were processed in Adobe Photoshop using gamma adjustments to enhance visibility of intermediate gray values and median filtering to suppress noise when necessary.

### Tissue Clearing and Imaging of Cleared Tissue

**Figure 1:**
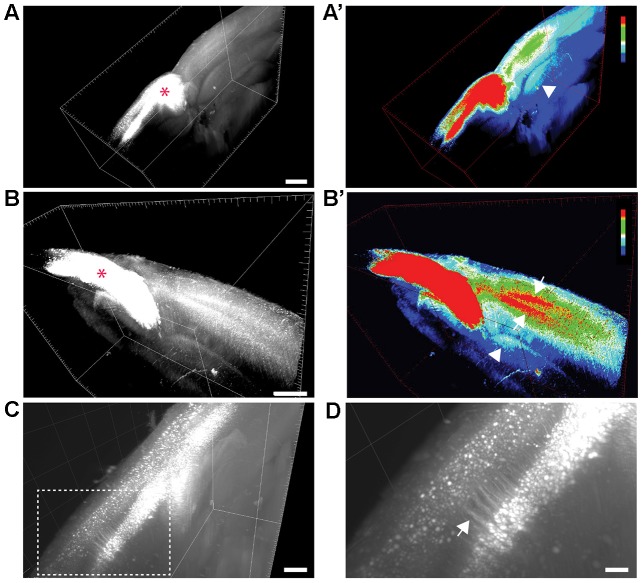
**Visualization of transcallosal projections in 2 mm thick cleared brain section using 3DISCO clearing. (A,B)** 3D-views of thick cleared brain sections showing the distribution of transcallosal projections throughout the brain section using two side views (**A,B**; red asterisk shows the injection site). **(A’,B’)** 3D-HeatMap views (generated in ImageJ/Fiji) identical to the cleared brains presented in **(A,B)** showing the intensity of labeling and the distribution of transcallosal projections contralateral to the injection (intensity scale from blue: low intensity to red: high intensity created using the ImageJ plugin HeatMap Histogram). Note in **(A’)** the axonal path of transcallosal neurons indicated with a white arrowhead and in **(B’)** projection to two distinct areas in the contralateral hemisphere (white arrows). **(C)** Higher magnification 3D-view of cleared thick brain section showing apical dendrites of layer V transcallosal neurons at the injection site. **(D)** Magnification (5×) of the box image in **(C)**. Scale bars equal 1 mm in **(A,A’)**, 500 μm in **(B,B’)** and **(C)**, 100 μm in **(D)**.

Transcallosal connections were traced using an AAV-EYFP (see above for production and titer). Traced brains were cleared using a simplified version of the 3DISCO protocol first described by Ertürk et al. (2012). All clearing steps were performed at room temperature. For the first step of clearing, 50% (vol/vol), 70% and 80% of Tetrahydrofuran/H_2_O (THF; Sigma 186562-12X100ML) dilutions in distilled water were prepared. Samples were first incubated with 50% THF for 1 h, followed by 70% and 80% THF for 1 h, each in a glass vial on a rotor. The sample bottles were covered with aluminum foil and incubation was carried out in the dark to avoid bleaching. Samples were then incubated with 100% THF for 1 h, overnight (o/n) and again for 1 h followed by incubation in Dichloromethane (DCM; Sigma 270997-12X100ML) for at least 1 h or until they sank to the bottom of the vial. Finally, samples were incubated in Dibenzyl Ether (DBE; Sigma 108014-1KG) for at least 30 min or until clear (~1 h) and then stored in DBE at room temperature. Note that the samples were immersed completely in each clearing solution in an eppendorf tube (~4–5 ml) filled up to the brim to avoid any air bubbles in the tube. Cleared samples were kept in the final clearing solution (DBE) including during imaging. Thick brain sections, (2 mm) were imaged on a light-sheet microscope imaging system (UM II LaVision BioTec-UltraMicroscope II) equipped with a 4× objective (Olympus XLFLURO4x), with 0.28 NA and a lens equipped with a 10 mm working distance dipping cap and *z*-step size of 3 μm. For the images presented on Figure 1, 3D-projections were performed with Imaris (Bitplane^1^). HeatMap Histograms were generated in Fiji/ImageJ (Schindelin et al., 2012).

### Data Analysis

#### Quantification of the Location of the Terminal Fields of Transcallosal Axons Following Anterograde Labeling

BDA 10,000 MW was injected either in layers II/III or layer V of the cortex in order to determine whether transcallosal neurons located in different cortical layers have differential projections in the contralateral homotopic column. The distribution of transcallosal axons in the contralateral cortex was quantified on 3–5 sections per animal in Fiji/ImageJ (Schindelin et al., 2012). The sections were selected relative to the distance from the point of injection, starting from section zero at the center of injection (−1.5 mm from Bregma) and the two immediate sections rostrally and caudally to section zero. Cortical layers were defined based on NeuroTrace staining. The extent of the area in which the terminal fields are located was circled per layer and across the entire depth of the cortex. Then, integrated densities were calculated using the “measure tool” in Fiji/ImageJ for each layer as well as across the depth of the cortex (total integrated density). Integrated density is defined as the product of the area analyzed by the mean intensity in this area (the sum of the intensity values of all the pixels in the selection divided by the number of pixels). To calculate the proportion of terminal fields in a given contralateral cortical layer, the integrated fluorescence intensities of this layer were determined using the “measure tool” in Fiji/ImageJ and related to the total integrated fluorescence intensity measured throughout the cortical column.

#### Quantification of Transcallosal Projection Neurons Following Retrograde Labeling with FluoroGold

For the analysis of the FG-labeled transcallosal neurons, five sections per animal were selected and imaged using an Olympus FV1000 confocal microscope. The selection of the sections was based on the distance from the point of injection. First, we defined the center of injection (section zero) as the section which had the most intense labeling on the ipsilateral side and corresponded to Bregma −1.5 mm for the group injected caudal to Bregma and Bregma +0.3 mm for the group injected rostral to Bregma. According to that, we imaged a total of five sections (including the section directly homotopic to the center of injection), corresponding to the following coordinates: −2.1 mm, −1.8 mm, −1.5 mm, −1.2 mm, −0.9 mm from Bregma (for the injections caudal to Bregma) and −0.7 mm, 0 mm, +0.3 mm, +0.6 mm, +0.9 mm from Bregma (for the injections rostral to Bregma), respectively. Quantification of the retrogradely-labeled FG positive cell bodies of transcallosal projections was carried out on a confocal maximum intensity projection (*z*-stack). FG labeled cells were counted manually using the cell-counter plugin of Fiji/Image-J. A total of three areas—contralateral to the hemisphere of injection—were selected to analyze the distribution of FG positive neurons: the homotopic area (HA), the center of which was positioned at the same distance from the midline as the center of the injection site and the lateral adjacent area (LAA) and medial adjacent area (MAA). The size of each analyzed area in the three locations was 1226 mm^2^. To analyze the distribution of the cells across the areas, cells in each area were counted separately and related to the total number of cells present in all three areas (HA + LAA + MA) of the same section.

In order to define the cortical layers, in which these neurons were located we used NeuroTrace labeling. Then, we counted the number of FG positive cells in a given cortical layer of a given area (HA, MAA or LAA) and divided it by the total number of FG positive cells present in the entire area (HA, MAA or LAA).

To analyze the data, the results of the quantifications were first pooled across the different injection coordinates to give an overview of the general distribution of transcallosal projection neurons (as shown in Figures 3, 5). In order to determine the regional variability of this distribution between subpopulations of transcallosal neurons, data for all independent injection coordinates of the primary motor cortex and primary somatosensory cortex (outside and within the barrel cortex) were also analyzed individually (as shown in Figures 4, 6).

#### Statistical Evaluation

Results are given as mean ± SEM. GraphPad Prism 5.01 for Windows (GraphPad Software) was used to perform statistical analysis. For multiple comparisons a one-way analysis of variance (ANOVA) followed by a Tukey’s *post hoc* test was performed or a two-way ANOVA followed by Bonferroni *post hoc* test. Significance levels are indicated as follows: *p < 0.05; **p < 0.01; ***p < 0.001.

## Results

To study the organization of transcallosal connections in the mouse cortex we used both anterograde and retrograde tracing approaches. Anterograde tracing was employed to: (i) reconstruct the large-scale distribution of transcallosal axons in cleared brain tissue; and (ii) to investigate the layer-specific projection pattern of these axons in the contralateral cortex. Retrograde tracing of transcallosal neurons was used to determine the location of transcallosal projection neurons across a large part of the cortex covering motor and somatosensory coordinates. The overriding aim of this study was to reveal the regional variability of transcallosal connectivity in anatomically and functionally distinct brain regions.

### Anterograde Labeling and Brain Clearing Visualize the Organization of Transcallosal Connections *In Situ*

We used a brain clearing technique in order to visualize labeled transcallosal projections within the intact brain tissue and investigate the pattern of connections in the contralateral cortex in three dimensions (Figure 1). To ensure that enough transcallosal neurons were labeled and their corresponding axons could be imaged through a thick (2 mm) cleared brain we used a recombinant AAV, expressing EYFP under the control of the CAG promoter. Using 3DISCO clearing (Ertürk et al., 2012; Ertürk and Bradke, 2013; Renier et al., 2014), we could already detect the labeled projection neurons and their axonal projections in the contralateral cortex, at a low magnification (Figures 1A–C). The homotopic organization of transcallosal neurons was immediately apparent in the cleared tissue (Figures 1A–B′) a and transcallosal axons could be followed through the corpus callosum to the contralateral side (Figures 1A–B′; white arrowheads). Their transcallosal axons mainly projected to two distinct cortical layers (Figures 1B,B′; white arrows). Finally, the relatively sparse labeling achieved in our study allowed us to distinguish not only individual projection neurons in the cortex but also single neurites (Figures 1C,D), underscoring the capability of tissue clearing techniques to reveal the anatomy of defined axonal tract systems across different scales.

### Anterograde Labeling Reveals that Transcallosal Neurons Located in Layer II/III and Layer V of the Cortex Show Similar Innervation of the Entire Contralateral Cortical Column

**Figure 2:**
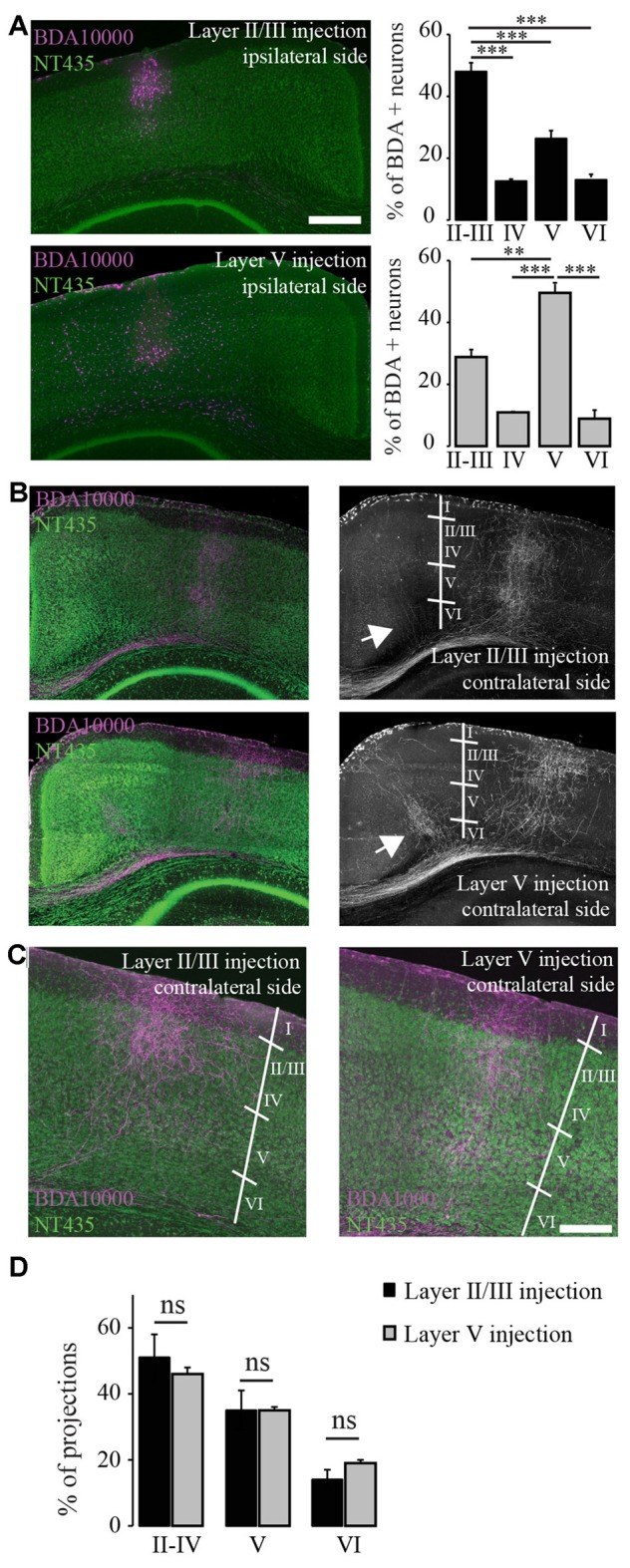
**Terminal fields of transcallosal axonal projections are located in distinct layers of the homotopic contralateral cortex with a specific targeting across cortical layers. (A)** Left: confocal images of transcallosal projections in the contralateral hemisphere following injection of the anterograde tracer biotinylated dextran amine (BDA) 10,000 MW in layer II/III (top) and in layer V (bottom) of the ipsilateral cortex (NeuroTrace, green; BDA 10,000 MW, purple). Right: quantification of the neurons labeled with BDA 10,000 MW in the different cortical layers following injections in layer II/III (top) and layer V (bottom). **(B)** Confocal images of transcallosal projections in the contralateral hemisphere following injection of the anterograde tracer BDA 10,000 MW in layer V of the ipsilateral cortex (NeuroTrace, green; BDA 10,000 MW, purple). White arrows in right images show the presence of axonal projections outside of the homotopic cortical area following injections in layer V of the cortex and to lesser degree following injections in layer II/III of the cortex. **(C)** Higher magnification of the contralateral cortex showing the terminal fields of transcallosal axons following injection in layer II/III (left panel) or layer V (right panel). **(D)** Quantification of the location of transcallosal axon terminals in the contralateral cortex and their repartition per cortical layer. No differences were found following injections in layer II/III and layer V. Scale bars equal 500 μm in **(A,B)** and 200 μm in **(C)**. One-way analysis of variance (ANOVA) followed by Tukey *post hoc* test: ^***^p < 0.001; ^**^p < 0.01.

In this experiment we took advantage of the tracer BDA 10,000 MW in order to anterogradely label transcallosal connections. We first wondered whether transcallosal neurons located in layers II/III and layer V of the cortex, would differ in their innervation pattern of the contralateral side. In order to answer this question, we injected BDA either in layers II/III or V of the cortex (Figure 2A). To verify the specificity of injection, we determined the proportion of BDA positive neurons at the site of injection in each cortical layer, defined based on Neurotrace labeling (Figure 2A). We observed that injections in layer II/III led to a predominant location of labeled cells in the corresponding layer (*p* < 0.001) and similarly injections in layer V led to a predominant location of labeled cells in the corresponding layer (*p* < 0.01). We then analyzed the projection pattern of transcallosal neurons in the contralateral hemisphere (Figures 2B–D) and found that, following both types of injections the trancallosal axons projected primarily to the HA and showed a strong columnar organization (Figures 2B,C), with terminal fields of the axons spanning the entire extent of the cortical column (Figures 2B,C). Furthermore, we could find additional transcallosal axon branches that enter the cortex more medial and more lateral from the main columnar projections (white arrows, Figure 2B). These ectopic projections were more frequently detected following layer V injections, but were also present following layer II/III injections.

Finally, we mapped the terminal fields of transcallosal axons labeled by layer II/III and layer V injections by measuring the integrated fluorescence intensities in distinct cortical layers of the homotopic contralateral cortex. Overall, no differences between the different injection sites were found. Also, on average (average of all injections sites) 48.4 ± 3.4% of the total projections were in layers II-IV, 35.1 ± 2.8% in layer V and 16.4 ± 1.8% in layer VI (Figure 2D). This underlines that transcallosal neurons located in layer II/III and layer V follow common organization principles and show similar contralateral projection patterns. It is however important to stress that those projections are not confined to layers II-IV and V but are also present in layer VI underscoring that they span the entire extent of the cortical column (Figures 2C,D).

### Retrograde Labeling Confirms the Overall Homotopic Organization of Transcallosal Projection Neurons but also Reveals Distinct Heterotopic Connections

**Figure 3:**
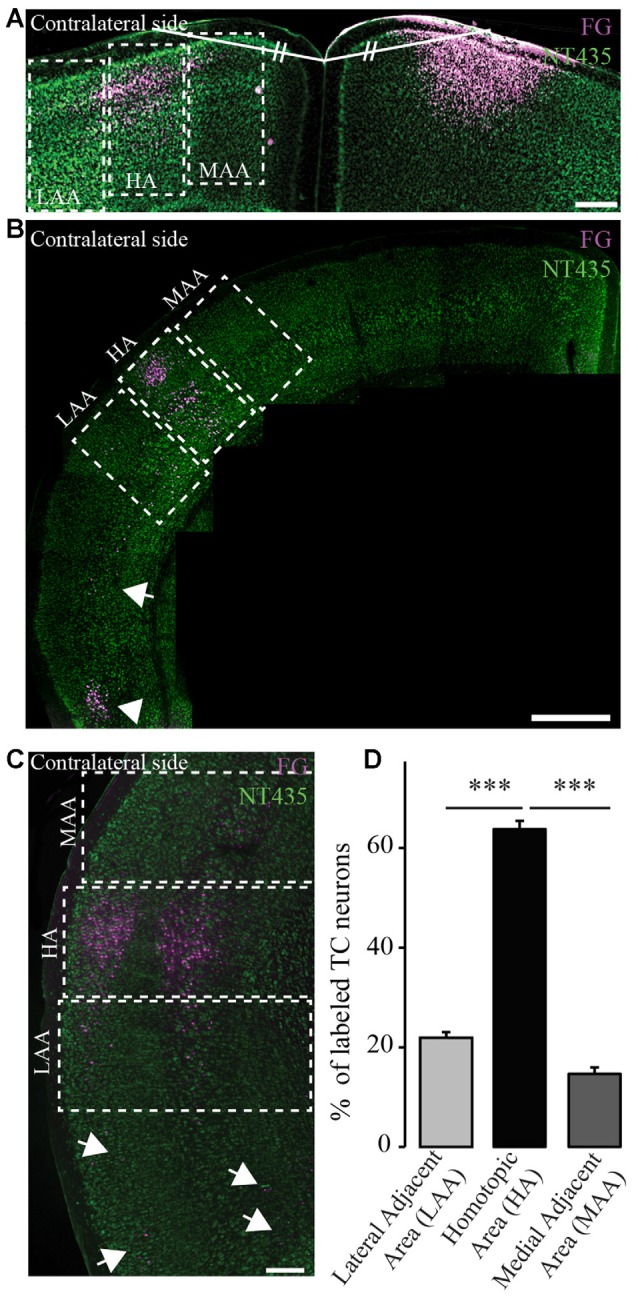
**Homotopic organization of transcallosal projection neurons.** **(A)** Confocal image of retrogradely labeled transcallosal projection neurons (injection in the motor cortex rostral to Bregma) that illustrates how we analyzed the homotopic organization of the transcallosal projection neurons. Shortly, a line was drawn from the center of injection of the retrograde tracer and the junction of the two hemispheres. Then a similar line (same length and same angle) was drawn to the contralateral side to define the homotopic area (HA), the lateral adjacent area (LAA) and the medial adjacent area (MAA). **(B,C)** Confocal pictures showing the organization of transcallosal projection neurons mostly in the area homotopic to the contralateral point of injection after injections in the barrel cortex caudal to Bregma **(B,C)**. The two adjacent areas show the presence of some projection neurons albeit in minor quantities. Some neurons were also found in the lower perirhinal cortex (**B**, white arrowhead and **C**, white arrows). **(D)** Quantification of the repartition of transcallosal projections neurons in the three areas defined in **(A)**. Scale bars equal 200 μm in **(A,B)** and 500 μm in **(C)**. One-way anova followed by Tukey *post hoc* test: ***p < 0.001.

In order to determine the location of the cell bodies of transcallosal axons we used FG as a retrograde tracer (Schmued and Fallon, 1986; Lanciego and Wouterlood, 2006). We used six different coordinates (spanning the primary motor cortex and primary somatosensory cortex). We found that most of the cells of origin of transcallosal axons are located in homotopic regions of the contralateral cortex. To assess this, we analyzed the following three areas for each injection: the area homotopic to the contralateral site of injection, as well as the similar-sized medial and LAAs (Figure 3A). When we analyzed all animals together, our findings show that the majority of transcallosal neurons are strongly and significantly (*p* < 0.001) located in the HA (Figures 3B,C). Indeed 63.8 ± 9.3% of the total number of cells of origin of transcallosal axons are located in the HA. Importantly up to 21.9 ± 6.1% of cells of origin of transcallosal axons were found in the LAA and up to 14.7 ± 7.2% in the MAA (Figure 3D) which underscores the relative importance of those heterotopic connections. It is, in addition, worth noting that some cells are found ectopically outside of the three above defined areas (Figures 3B,C; white arrows) in particular around the perirhinal cortex (Figure 3B; white arrowhead).

**Figure 4:**
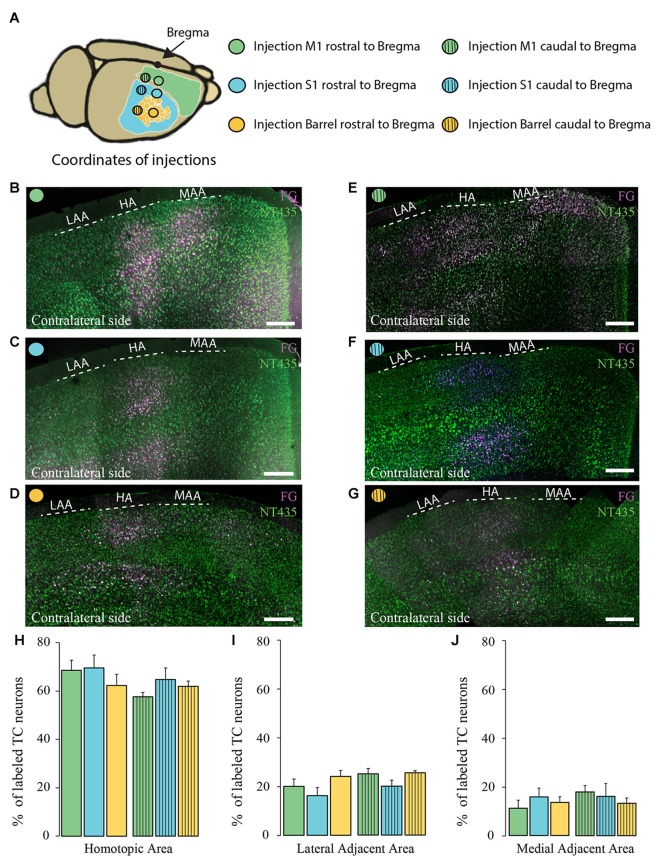
**Comparison of the homotopic organization of transcallosal projection neurons across different brain regions. (A)** Scheme of the stereotactic coordinates used for retrograde tracing in the study. **(B–G)** Confocal images of the contralateral cortex following injection of a retrograde tracer in motor cortex (rostral to Bregma; **B**), somatosensory cortex (rostral to Bregma; **C**), barrel cortex (rostral to Bregma; **D**), motor cortex (caudal to Bregma; **E**), somatosensory (caudal to Bregma; **F**), barrel cortex (caudal to Bregma; **G**) showing the homotopic organization of transcallosal projection neurons. **(H–J)** Quantification of the repartition of the transcallosal projection neurons in the contralateral cortex in the HA **(H)**, the LAA **(I)** and the MAA **(J)**. Full bars, injections in the cortex rostral to Bregma; dashed bars, injections in the cortex caudal to Bregma; green: injection in the motor cortex, blue: injection in the somatosensory cortex and yellow: injection in the barrel cortex. Scale bars equal 250 μm in **(B–G)**. One-way Anova followed by Tukey test were used for each area.

Next, we separately analyzed animals injected retrogradely in the primary motor cortex and in the primary somatosensory cortex (outside and within the barrel cortex). We observed that the homotopic organization of transcallosal neurons is mostly maintained independently of the site of injection (Figures 4A–J). For example, 68.5 ± 4.2% of the transcallosal neurons traced from the M1 cortex (rostral coordinate) were localized in the HA as well as 69.4 ± 5.3% of the neurons traced from the S1 cortex (rostral coordinate), 62.2 ± 4.7% of the neurons traced from the barrel cortex (rostral coordinate), 57 ± 1.7% of the neurons traced from the M1 cortex (caudal coordinate), 63 ± 4.8% traced from the S1 cortex (caudal coordinate) and 61 ± 2.2% barrel cortex (caudal coordinate; Figure 4H). Likewise, the percentages of transcallosal neurons located in the lateral adjacent and MAAs were in a similar range across all coordinates investigated in this study confirming that while the homotopic organization principle is maintained throughout the cortex, there is a significant proportion of transcallosal neurons that are located heterotopically (Figures 4I,J).

### Transcallosal Neurons throughout the Cortex Are Primarily Located in Layer II/III and Layer V but Can also be Found in Ectopic Layers Particularly in Heterotopic Areas

**Figure 5:**
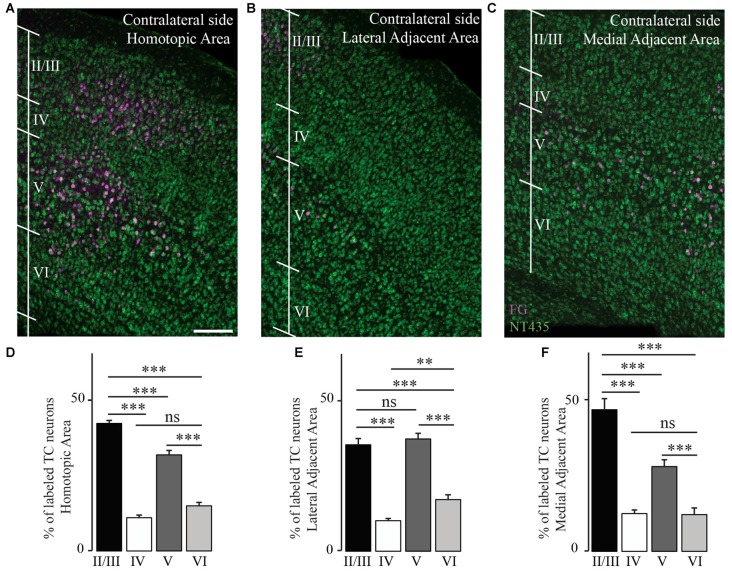
**Transcallosal projection neurons are primarily located in layer II/III and layer V of the contralateral cortex.** **(A)** Confocal image of the homotopic cortical area (HA) showing the presence of labeled transcallosal projection neurons in layer II/III and layer V. **(B)** Confocal image of the cortex focusing on the lateral adjacent projection area (LAA) showing similar numbers of transcallosal projection neurons in layer II/III and layer V. **(C)** Confocal image of the cortex focussing on the medial lateral adjacent projection area (MAA) showing the location of transcallosal projection neurons in layer II/III and layer V. **(D)** Quantification of the repartition of transcallosal projection neurons across different cortical layers in the HA. **(E)** Quantification of the repartition of transcallosal projection neurons across different cortical layers in LAA. **(F)** Quantification of the repartition of transcallosal projection neurons across different cortical layers in MAA. Scale bars equal 200 μm in **(A–C)**. One-way-Anova followed by Tukey *post hoc* test: ***p < 0.001; **p < 0.01.

We then investigated the distribution of transcallosal projection neurons across different cortical layers in the HA as well as in the adjacent lateral and medial regions. As previously, we relied on NeuroTrace staining to distinguish layers in the cortex and counted all neurons labeled with FG in the respective layers (Figures 5A–C). Analysis of all animals as a single group showed that transcallosal projection neurons in the HA are significantly and predominantly located in layer II/III (42.3 ± 1.0% of FG labeled neurons) and in layer V (31.8 ± 1.4% of FG labeled neurons). However smaller proportions of FG labeled cells—still averaging more than 20% of the total labeled neurons—were located in layer IV (11 ± 0.9%) and in layer VI (14.9 ± 1.2%; Figures 5A,D). These percentages changed slightly when considering the adjacent areas, in which the layer specific organization appears to be overall less strict, in particular in the LAA (Figures 5B–F).

**Figure 6:**
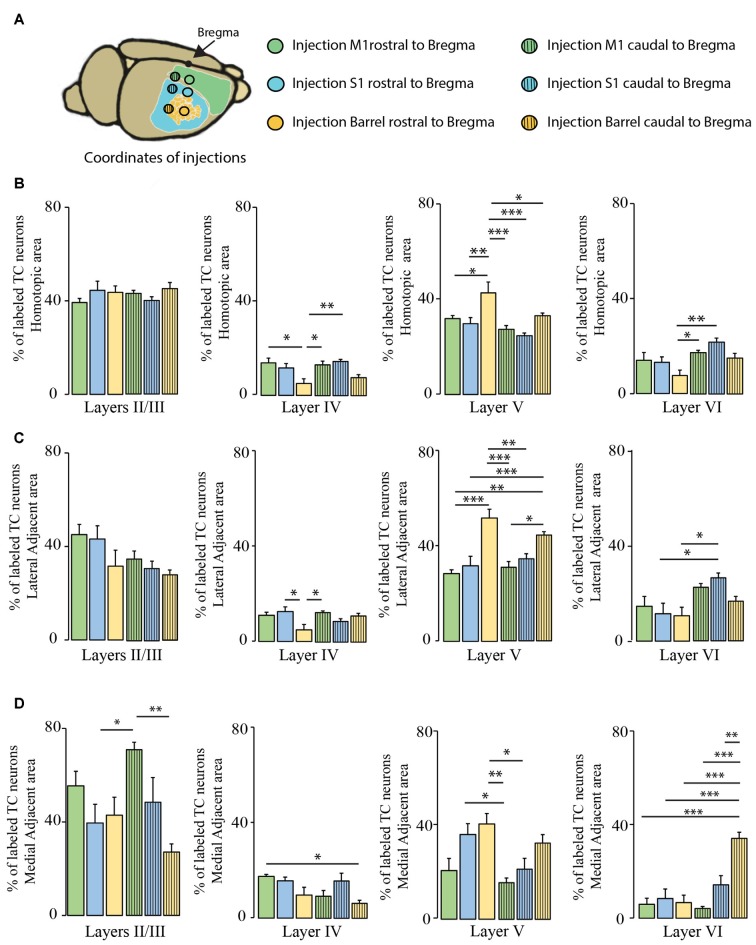
**Comparison of layer-specific organization of transcallosal projection neurons across the mouse cortex.** **(A)** Scheme of the stereotactic coordinates used for retrograde tracing in the study. **(B)** Quantification of the location of transcallosal neurons in different cortical layers of the HA following injections in the cortex rostral to Bregma (full bars) and caudal to Bregma (dashed bars). **(C)** Quantification of the location of transcallosal neurons in different cortical layers in the LAA following injections in the cortex rostral to Bregma (full bars) and caudal to Bregma (dashed bars). **(D)** Quantification of location of transcallosal neurons in different cortical layers in the MAA following injections in the cortex rostral to Bregma (full bars) and caudal to Bregma (dashed bars). Green: injection in the motor cortex, blue: injection in the somatosensory cortex and yellow: injection in the barrel cortex. Two way- Anova (variables: (i) Areas of labeling and (ii) Injection sites) followed by Bonferroni *post hoc* test performed for each independent dataset. ***p < 0.001; **p < 0.01; *p < 0.05.

We then investigated whether the distribution of the transcallosal projection neurons across cortical layers depends on the particular region of the cortex that was injected (Figure 6). In the HA, we found that most of the neurons were located in layer II/III of the cortex and in layer V, irrespective of the site of injection (Figures 6B–D). When we observed the LAA, our results showed that injection in the motor and somatosensory cortices revealed a similar distribution to that found in the HA with small marginal differences (Figures 6B,D). However, the injections into both regions of the barrel cortex showed that more transcallosal neurons are located in layer V than in layer II/III (Figure 6C). In contrast, in the MAA in particular of the primary motor cortex a comparably high proportion of transcallosal neurons are located in layer II/III (more than 50% for the injections rostral to Bregma and more than 70% for the injections caudal to Bregma; Figures 5B,C, 6D). Taken together these results confirm a rather uniform organization of transcallosal connections. This organization appears to be particular homogenous in the HA and shows more regional variability in the adjacent medial and lateral areas.

## Discussion

In this study we have used anterograde and retrograde tracing techniques to perform a systematic analysis of transcallosal axonal pathways across the motor and somatosensory cortex. The aim of the study was to determine whether transcallosal pathways are anatomically organized in a uniform fashion or whether differences between functionally distinct brain regions can be detected. Our conclusion is that, overall, transcallosal projections are uniformly organized across the cerebral cortex. This organization pattern is particularly rigorous in the HA, while some degree of regional variability is detected in the adjacent medial and lateral areas.

### Visualization of the Transcallosal Axonal Pathway in Cleared Brain Tissue

In order to visualize the transcallosal pathways throughout an entire cleared brain block we used the 3DISCO procedure (Ertürk et al., 2012; Ertürk and Bradke, 2013). Although we used a recombinant AAV with a strong promoter driving the yellow fluorescent protein to label the axonal tract anterogradely (EYFP), we found that the tissue had to be imaged rapidly to conserve the quality of the fluorescence as the clearing solution degrades the fluorescent signal over time as noticed before Ertürk et al. (2012). When we imaged the samples shortly after completion of the clearing procedure, we found that transcallosal connections can be visualized in intact cleared brain blocks without further immunostaining. Not only could we detect the site of injection but also the contralateral projection sites as well as the cortical layers in which the transcallosal axons terminate. The degree of resolution was such that we could, when labeling was sparse, also detect single neurites of transcallosal projection neurons, indicating that clearing techniques can be used to reconstruct fluorescently labeled transcallosal projections (Renier et al., 2014).

### Organization of Transcallosal Neurons and Their Projections

We used conventional anterograde tracing techniques to further characterize the projection pattern of transcallosal neurons in the contralateral cortex. We first investigated where the axonal fields terminated, depending on the depth of injection in the ipsilateral cortex. We performed this experiment in order to determine whether transcallosal neurons, located in layers II/III or layer V, project their axons to different cortical locations. While several previous reports have mentioned that transcallosal neurons predominantly reside in layer II/III (Wise and Jones, 1976; Peters et al., 1990; White and Czeiger, 1991; Fame et al., 2011) others also found a significant amount of the neurons in layer V (O’Leary et al., 1981). Based on these previous observations we chose to inject the anterograde tracer alternatively in layers II/III and in layer V. We found that neither the homotopic organization nor the distribution of axon terminals across different cortical layers is affected by the site of injection. It is however important to note that the transcallosal projections show columnar organization with terminal axons spanning the entire extent of the cortical column. This indicates that both subpopulations of transcallosal neurons—whether they reside in deep layers of the cortex or in superficial layers of the cortex—maintain similarly organized projections to the contralateral hemisphere and that their projections through the cortical layers might be more widely spread out than previously reported. With regards to our experiment it is important to note, that we injected relatively high amounts (1.5 μl) of BDA tracer, in order to label enough transcallosal neurons and obtain a good visualization of the terminal field distribution. While this approach led to some spread of the tracer only in immediately adjacent cortical layers (e.g., layer I and IV for layer II/ III injections and layer IV and VI for layer V injections) we are confident that this did not affect our conclusion as both types of injections resulted in essentially the same projection pattern while the primary ipsilateral labeling was strongly localized to the injected layers (Figure 2A) Interestingly, injections in layer V also gave rise to a slightly higher number of ectopic (outside of the homotopic region) axon terminal fields when compared to injections in layers II/ III. These ectopic projections might have a specific significance in inter-hemispheric connectivity.

In order to determine the organization of transcallosal projection neurons we used the retrograde tracer FG to investigate the location of transcallosal projection neurons in the contralateral cortex. We found that about 2/3rd of all neurons retrogradely labeled are localized in a region homotopic to the injection area, which is in agreement with previous reports (Schüz et al., 2006; Fame et al., 2011; Oswald et al., 2013), emphasizing that a sizeable proportion of those transcallosal projection neurons resides in heterotopic areas. When we further assessed the distribution of those projection neurons across the different cortical layers we found that most of these neurons are located in layers II/III and layer V of the cortex. This finding is in accordance with previous reports (Wise and Jones, 1976; O’Leary et al., 1981; Peters et al., 1990; White and Czeiger, 1991; Fame et al., 2011) that either showed predominant presence in layer II/III (Fame et al., 2011) or in layer V (O’Leary et al., 1981). While previous reports however often suggest an almost exclusive localization of transcallosal neurons within layer II/III and V (Fame et al., 2011), we observed that about 20% of these neurons are located in layers IV and VI. A similar distribution with a preferential but not exclusive localization of transcallosal neurons in layers II/III and V was also found when we analyzed the two areas adjacent to the HA (which encompasses fewer labeled cells but still about 15%–20% of the transcallosal neurons). This new finding is important as it underscores the importance of these heterotopic connections in normal mice. As it is reported that heterotopic connections increase following partial callosal agenesis (Wahl et al., 2009), it is possible that these connections are important for compensatory behavioral and cognitive function following innate or acquired callosal defects.

### Regional Commonalities and Differences of Transcallosal Projections Neurons Across the Cortex

So far, the majority of studies, which investigated the organization of transcallosal neurons, have only done so at a single brain region, most often focusing on the somatosensory cortex (Wise and Jones, 1976; Peters et al., 1990; White and Czeiger, 1991; Schüz et al., 2006). Directly comparing these studies is difficult as they often used different tracing and evaluation approaches. Here we wanted to study whether transcallosal projections are stereotypically organized across different brain regions or whether they show substantial structural adaptations in anatomically and functionally distinct cortical areas. This determines amongst others to what extent findings about transcallosal connectivity in one region can be transferred to other parts of the cortex. To assess the regional diversity we therefore used standardized tracing approaches to reveal the organization of transcallosal connections in six distinct brain areas covering a wide range of coordinates of the primary somatosensory and primary motor cortex. Our results show that in all cortical regions investigated transcallosal connections are structured remarkably similar both with regards to the homotopy of their projections and the layer-specific organization of the projection neurons. In this context it is interesting to note that some transcallosal neurons—in particular those located in the deeper cortical layers can have dual axonal projections (Cauller et al., 1998; Mitchell and Macklis, 2005; Wahl and Ziemann, 2008). Indeed both our anterograde and retrograde analysis also identified a smaller proportion of heterotopic connections that often targeted adjacent cortical areas.

While the overall organization of transcallosal connections is thus uniform across the different cortical areas, we also found important regional differences. For example, the distribution of transcallosal neurons across cortical layers seems to depend on their location in the primary somatosensory or the primary motor cortex. Interestingly such regional differences are primarily observed in heterotopically projecting neurons, while the organization of homotopically projecting neurons shows substantially less variability. This underlines the idea that these heterotopically projecting neurons might serve different functional purposes in the primary motor and primary somatosensory cortex. One option is that these connections can be recruited as part of a compensatory reorganization of neuronal connections following innate callosal agenesis or postnatal injury.

As correct inter-hemispheric connectivity is crucial for a precise cortical processing and integration it will thus be important to further understand possibly distinct functional properties of homotopic and heterotopic transcallosal projection neurons in both motor and sensory function. We hope that the systematic characterization of transcallosal connections that we provide here will help to address such questions in the future and allow us to better understand how inter-hemispheric connectivity is first established, modified by different functional requirements and altered by pathological processes.

## Author Contributions

FMB conceived the experiments. AC and LE performed surgeries, tracing, staining and imaging and analyzed the experiments. DC performed tissue clearing experiments from surgery to analysis. FMB, AC, LE and DC wrote the article. All authors contributed to the manuscript. AC and LE contributed equally to the manuscript and are co-first authors.

## Conflict of Interest Statement

The authors declare that the research was conducted in the absence of any commercial or financial relationships that could be construed as a potential conflict of interest.

## Footnotes

^1^http://www.bitplane.com/imaris/imaris

## References

[B1] Aboitiz F. Montiel J. (2003). One hundred million years of interhemispheric communication: the history of the corpus callosum. Braz. J. Med. Biol. Res. 36, 409–420. 10.1590/s0100-879x2003000400002 12700818

[B2] Bareyre F. M. Haudenschild B. Schwab M. E. (2002). Long-lasting sprouting and gene expression changes induced by the monoclonal antibody IN-1 in the adult spinal cord. J. Neurosci. 22, 7097–7110. 12177206 10.1523/JNEUROSCI.22-16-07097.2002 PMC6757902

[B3] Bareyre F. M. Kerschensteiner M. Raineteau O. Mettenleiter T. C. Weinmann O. Schwab M. E. (2004). The injured spinal cord spontaneously forms a new intraspinal circuit in adult rats. Nat. Neurosci. 7, 269–277. 10.1038/nn1195 14966523

[B4] Cauller L. J. Clancy B. Connors B. W. (1998). Backward cortical projections to primary somatosensory cortex in rats extend long horizontal axons in layer I. J. Comp. Neurol. 390, 297–310. 10.1002/(SICI)1096-9861(19980112)390:2<297::AID-CNE11>3.3.CO;2-0 9453672

[B5] Egaas B. Courchesne E. Saitoh O. (1995). Reduced size of corpus callosum in autism. Arch. Neurol. 52, 794–801. 10.1001/archneur.1995.00540320070014 7639631

[B6] Ertürk A. Becker K. Jährling N. Mauch C. P. Hojer C. D. Egen J. G.. (2012). Three-dimensional imaging of solvent-cleared organs using 3DISCO. Nat. Protoc. 7, 1983–1995. 10.1038/nprot.2012.119 23060243

[B7] Ertürk A. Bradke F. (2013). High-resolution imaging of entire organs by 3-dimensional imaging of solvent cleared organs (3DISCO). Exp. Neurol. 242, 57–64. 10.1016/j.expneurol.2012.10.018 23124097

[B8] Fame R. M. MacDonald J. L. Macklis J. D. (2011). Development, specification, and diversity of callosal projection neurons. Trends Neurosci. 34, 41–50. 10.1016/j.tins.2010.10.002 21129791 PMC3053014

[B9] Freitag C. M. Luders E. Hulst H. E. Narr K. L. Thompson P. M. Toga A. W.. (2009). Total brain volume and corpus callosum size in medication-naïve adolescents and young adults with autism spectrum disorder. Biol. Psychiatry 66, 316–319. 10.1016/j.biopsych.2009.03.011 19409535 PMC3299337

[B10] Garcez P. P. Henrique N. P. Furtado D. A. Bolz J. Lent R. Uziel D. (2007). Axons of callosal neurons bifurcate transiently at the white matter before consolidating an interhemispheric projection. Eur. J. Neurosci. 25, 1384–1394. 10.1111/j.1460-9568.2007.05387.x 17425565

[B11] Grimm D. Kay M. A. Kleinschmidt J. A. (2003). Helper virus-free, optically controllable, and two-plasmid-based production of adeno-associated virus vectors of serotypes 1 to 6. Mol. Ther. 7, 839–850. 10.1016/s1525-0016(03)00095-9 12788658

[B12] Jacobi A. Loy K. Schmalz A. M. Hellsten M. Umemori H. Kerschensteiner M.. (2015). FGF22 signaling regulates synapse formation during post-injury remodeling of the spinal cord. EMBO J. 34, 1231–1243. 10.15252/embj.201490578 25766255 PMC4426482

[B13] Jacobson S. Trojanowski J. Q. (1974). The cells of origin of the corpus callosum in rat, cat and rhesus monkey. Brain Res. 74, 149–155. 10.1016/0006-8993(74)90118-8 4211227

[B14] Klugmann M. Symes C. W. Leichtlein C. B. Klaussner B. K. Dunning J. Fong D.. (2005). AAV-mediated hippocampal expression of short and long Homer 1 proteins differentially affect cognition and seizure activity in adult rats. Mol. Cell. Neurosci. 28, 347–360. 10.1016/j.mcn.2004.10.002 15691715

[B15] Lanciego J. L. Wouterlood F. G. (2006). “Multiple neuroanatomical tract-tracing: approaches for multiple tract-tracing,” in Neuroanatomical Tract-Tracing 3. Molecules, Neurons, and Systems, eds Zaborszky L. Wouterlood F. G. Lanciego J. L. (New York, NY: Springer), 336–365.

[B16] Lang C. Bradley P. M. Jacobi A. Kerschensteiner M. Bareyre F. M. (2013). STAT3 promotes corticospinal remodelling and functional recovery after spinal cord injury. EMBO Rep. 14, 931–937. 10.1038/embor.2013.117 23928811 PMC3807223

[B17] McAlonan G. M. Cheung C. Cheung V. Wong N. Suckling J. Chua S. E. (2009). Differential effects on white-matter systems in high-functioning autism and Asperger’ syndrome. Psychol. Med. 39, 1885–1893. 10.1017/S0033291709005728 19356262

[B18] Mitchell B. D. Macklis J. D. (2005). Large-scale maintenance of dual projections by callosal and frontal cortical projection neurons in adult mice. J. Comp. Neurol. 482, 17–32. 10.1002/cne.20428 15612019

[B19] O’Leary D. D. Stanfield B. B. Cowan W. M. (1981). Evidence that the early postnatal restriction of the cells of origin of the callosal projection is due to the elimination of axonal collaterals rather than to the death of neurons. Brain Res. 227, 607–617. 10.1016/0165-3806(81)90012-2 7260661

[B20] Oswald M. J. Tantirigama M. L. S. Sonntag I. Hughes S. M. Empson R. M. (2013). Diversity of layer 5 projection neurons in the mouse motor cortex. Front. Cell. Neurosci. 7:174. 10.3389/fncel.2013.00174 24137110 PMC3797544

[B21] Paul L. K. Brown W. S. Adolphs R. Tyszka J. M. Richards L. J. Mukherjee P.. (2007). Agenesis of the corpus callosum: genetic, developmental and functional aspects of connectivity. Nat. Rev. Neurosci. 8, 287–299. 10.1038/nrn2107 17375041

[B22] Peters A. Payne B. R. Josephson K. (1990). Transcallosal non-pyramidal cell projections from visual cortex in the cat. J. Comp. Neurol. 302, 124–142. 10.1002/cne.903020110 2086610

[B23] Reiner A. Veenman C. L. Medina L. Jiao Y. Del Mar N. Honig M. G. (2000). Pathway tracing using biotinylated dextran amines. J. Neurosci. Methods 103, 23–37. 10.1016/s0165-0270(00)00293-4 11074093

[B24] Renier N. Wu Z. Simon D. J. Yang J. Ariel P. Tessier-Lavigne M. (2014). iDISCO: a simple, rapid method to immunolabel large tissue samples for volume imaging. Cell 159, 896–910. 10.1016/j.cell.2014.10.010 25417164

[B25] Schindelin J. Arganda-Carreras I. Frise E. Kaynig V. Longair M. Pietzsch T.. (2012). Fiji: an open-source platform for biological-image analysis. Nat. Methods 9, 676–682. 10.1038/nmeth.2019 22743772 PMC3855844

[B26] Schmued L. C. Fallon J. H. (1986). Fluoro-gold: a new fluorescent retrograde axonal tracer with numerous unique properties. Brain Res. 377, 147–154. 10.1016/0006-8993(86)91199-6 2425899

[B27] Schoenemann P. T. Sheehan M. J. Glotzer L. D. (2005). Prefrontal white matter volume is disproportionately larger in humans than in other primates. Nat. Neurosci. 8, 242–252. 10.1038/nn1394 15665874

[B28] Schüz A. Chaimow D. Liewald D. Dortenman M. (2006). Quantitative aspects of corticocortical connections: a tracer study in the mouse. Cereb. Cortex 16, 1474–1486. 10.1093/cercor/bhj085 16357338

[B29] Vidal C. N. Nicolson R. DeVito T. J. Hayashi K. M. Geaga J. A. Drost D. J.. (2006). Mapping corpus callosum deficits in autism: an index of aberrant cortical connectivity. Biol. Psychiatry 60, 218–225. 10.1016/j.biopsych.2005.11.011 16460701

[B30] Wahl M. Strominger Z. Jeremy R. J. Barkovich A. J. Wakahiro M. Sherr E. H.. (2009). Variability of homotopic and heterotopic callosal connectivity in partial agenesis of the corpus callosum: a 3T diffusion tensor imaging and Q-ball tractography study. Am. J. Neuroradiol. 30, 282–289. 10.3174/ajnr.A1361 19001538 PMC7051413

[B31] Wahl M. Ziemann U. (2008). The human motor corpus callosum. Rev. Neurosci. 19, 451–466. 10.1515/revneuro.2008.19.6.451 19317183

[B32] White E. L. Czeiger D. (1991). Synapses made by axons of callosal projection neurons in mouse somatosensory cortex: emphasis on intrinsic connections. J. Comp. Neurol. 303, 233–244. 10.1002/cne.903030206 2013638

[B33] Wise S. P. Jones E. G. (1976). The organization and postnatal development of the commissural projection of the rat somatic sensory cortex. J. Comp. Neurol. 168, 313–343. 10.1002/cne.901680302 950383

[B34] Yorke C. H. Jr. Caviness V. S. Jr. (1975). Interhemispheric neocortical connections of the corpus callosum in the normal mouse: a study based on anterograde and retrograde methods. J. Comp. Neurol. 164, 233–245. 10.1002/cne.901640206 1184784

[B35] Zhou J. Wen Y. She L. Sui Y. N. Liu L. Richards L. J.. (2013). Axon position within the corpus callosum determines contralateral cortical projection. Proc. Natl. Acad. Sci. U S A 110, E2714–E2723. 10.1073/pnas.1310233110 23812756 PMC3718183

